# Supplementary figures and images for: Dietary sodium chloride attenuates increased β-cell mass to cause glucose intolerance in mice under a high-fat diet
Source: PLoS One. 2021 Mar 17;16(3):e0248065. doi: 10.1371/journal.pone.0248065 (PMC7968668; doi:10.1371/journal.pone.0248065)

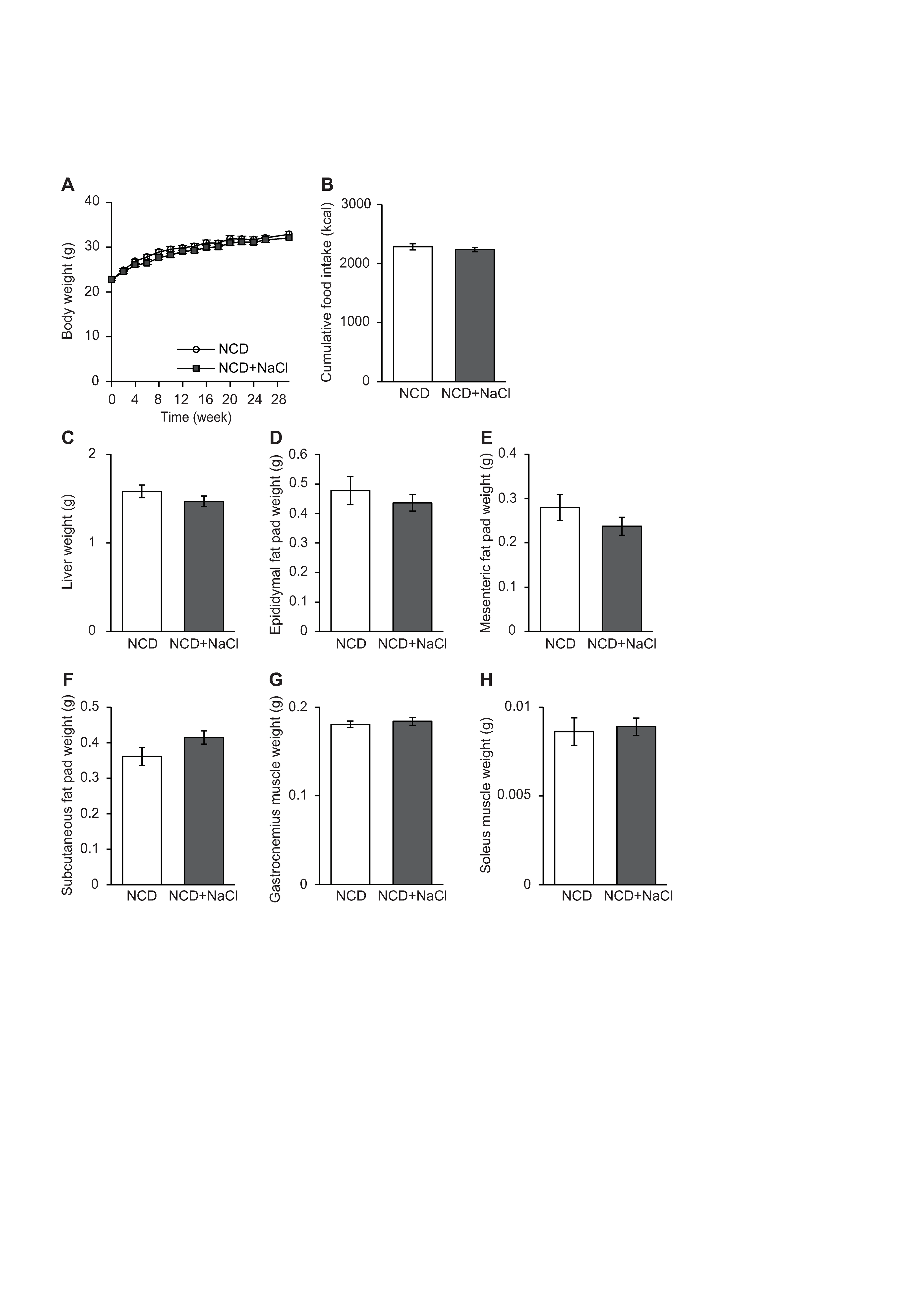

Supplement: S1 Fig — (A) Body weight and (B) cumulative food intake in mice fed a normal chow diet (NCD) or a NCD plus NaCl for 30 weeks (n = 16/group). (C) Liver, (D) epididymal fat pad, (E) mesenteric fat pad, (F) subcutaneous fat pad, (G) gastrocnemius muscle, and (H) soleus muscle weights in mice fed a NCD or a NCD plus NaCl for 30 weeks (n = 16/group). All values are mean ± SEM. (TIF) [file pone.0248065.s001.tif]

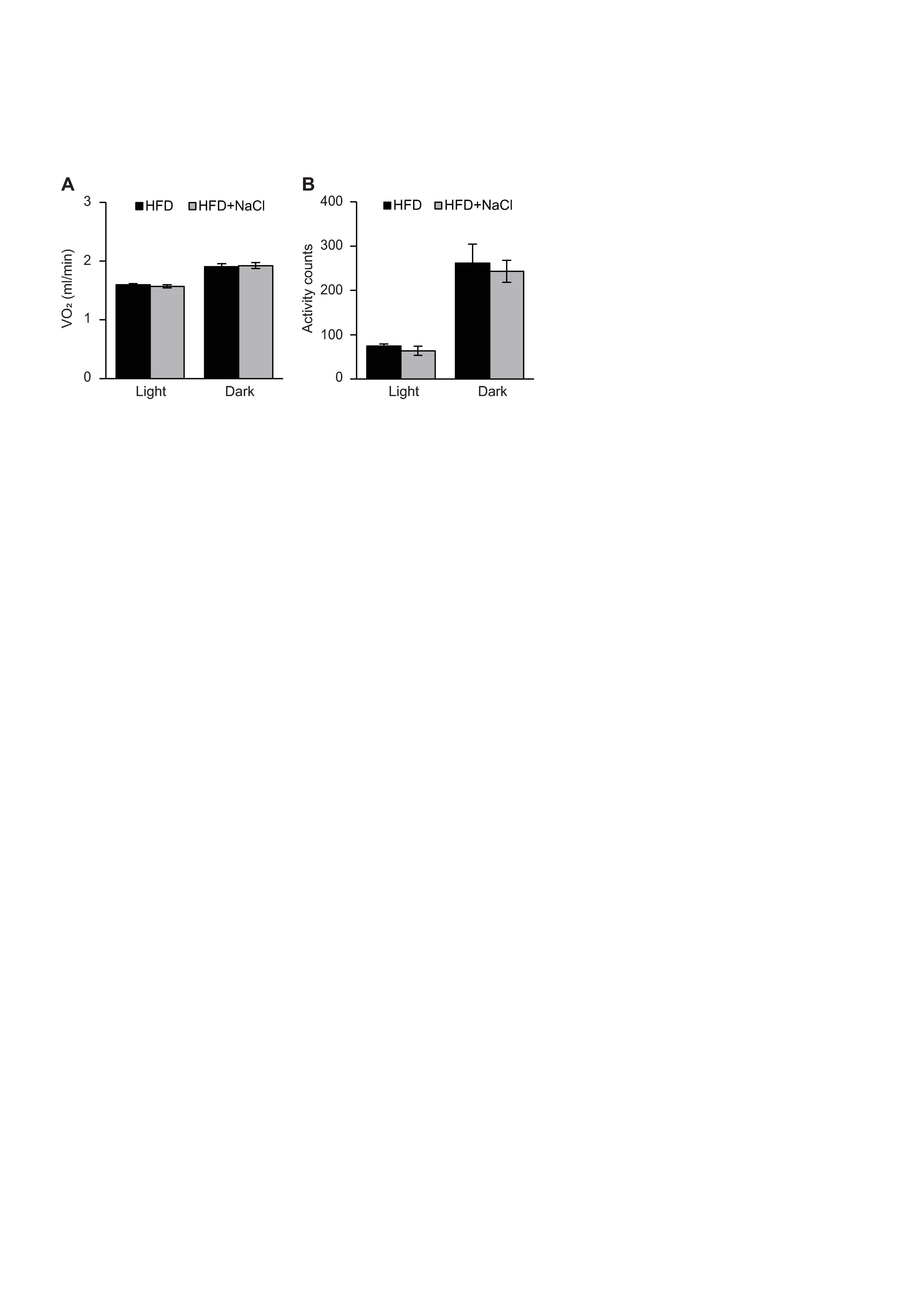

Supplement: S2 Fig — (A) Oxygen consumption (VO2) and (B) locomotor activity in mice fed a high-fat diet (HFD) or a HFD plus NaCl (n = 5/group). All values are mean ± SEM. (TIF) [file pone.0248065.s002.tif]

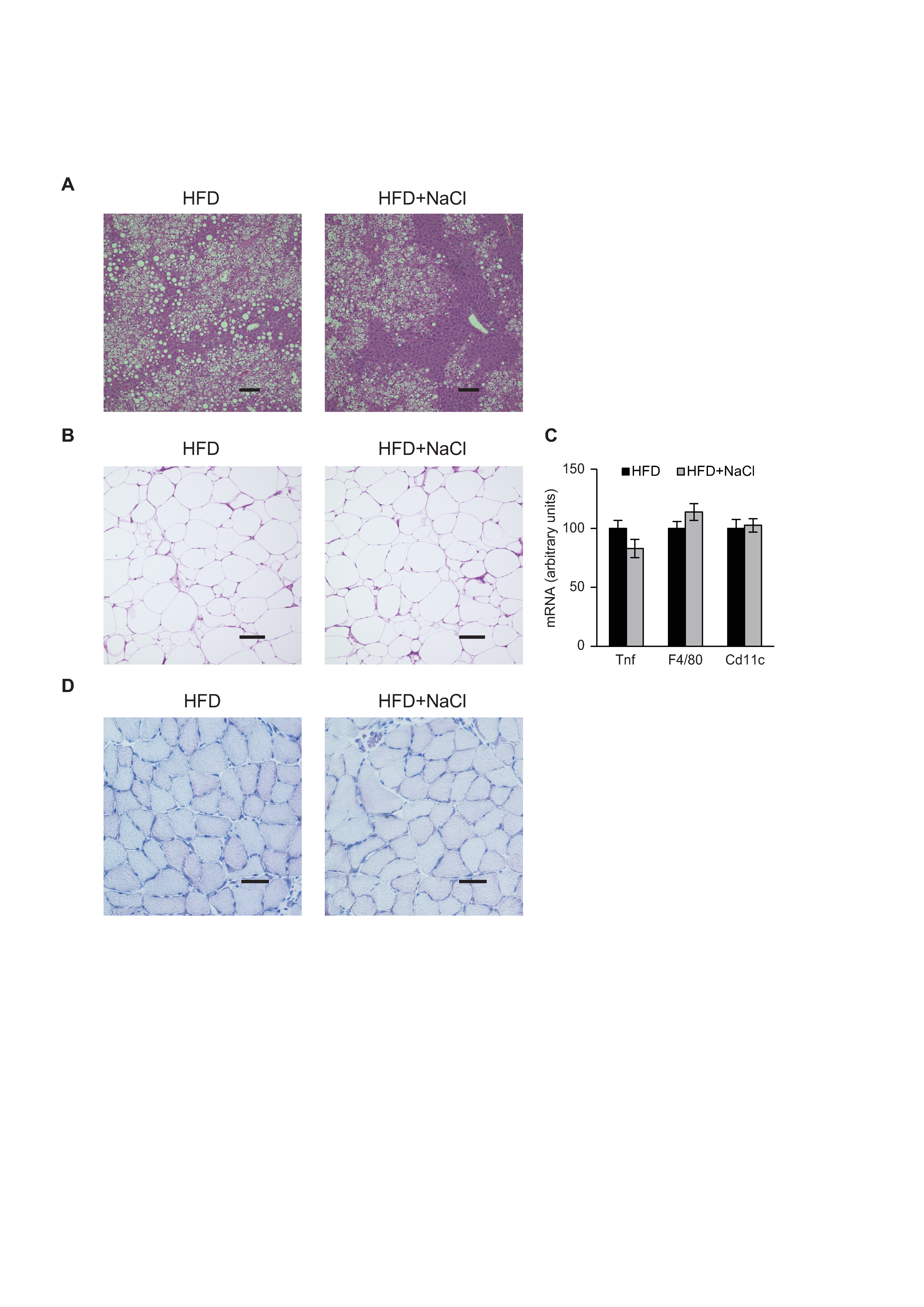

Supplement: S3 Fig — (A) Hematoxylin and eosin staining of liver sections from mice fed a high-fat diet (HFD) or HFD plus NaCl for 30 weeks. Scale bars, 100 μm. (B) Hematoxylin and eosin staining of epididymal white adipose tissue (WAT) in mice fed a HFD or HFD plus NaCl for 30 weeks. Scale bars, 100 μm. (C) Messenger RNA expression levels of TNF, F4/80, and Cd11c in mice fed a HFD or a HFD plus NaCl for 30 weeks as assessed by quantitative real-time PCR (n = 11–16/group). (D) Oil Red O staining of soleus from mice fed a HFD or HFD plus NaCl for 30 weeks. Scale bars, 50 μm. All values are mean ± SEM. (TIF) [file pone.0248065.s003.tif]

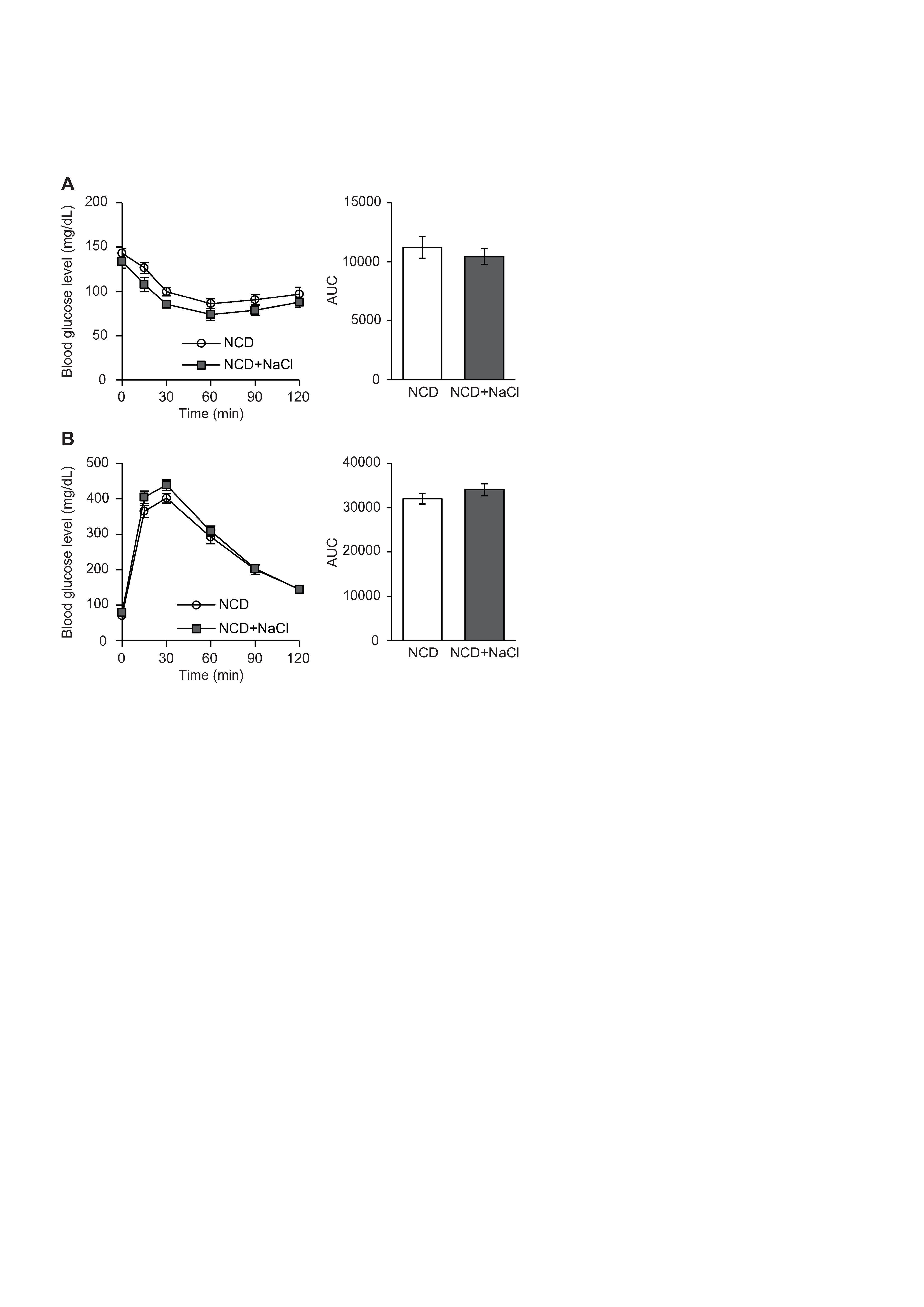

Supplement: S4 Fig — (A) An insulin tolerance test (ITT) and the area under curve (AUC) in mice fed a normal chow diet (NCD) or NCD plus NaCl for 25 weeks (n = 14–15/group). (B) A glucose tolerance test (GTT) and AUC in mice fed a NCD or NCD plus NaCl for 26 weeks (n = 13–16/group). All values are mean ± SEM. (TIF) [file pone.0248065.s004.tif]

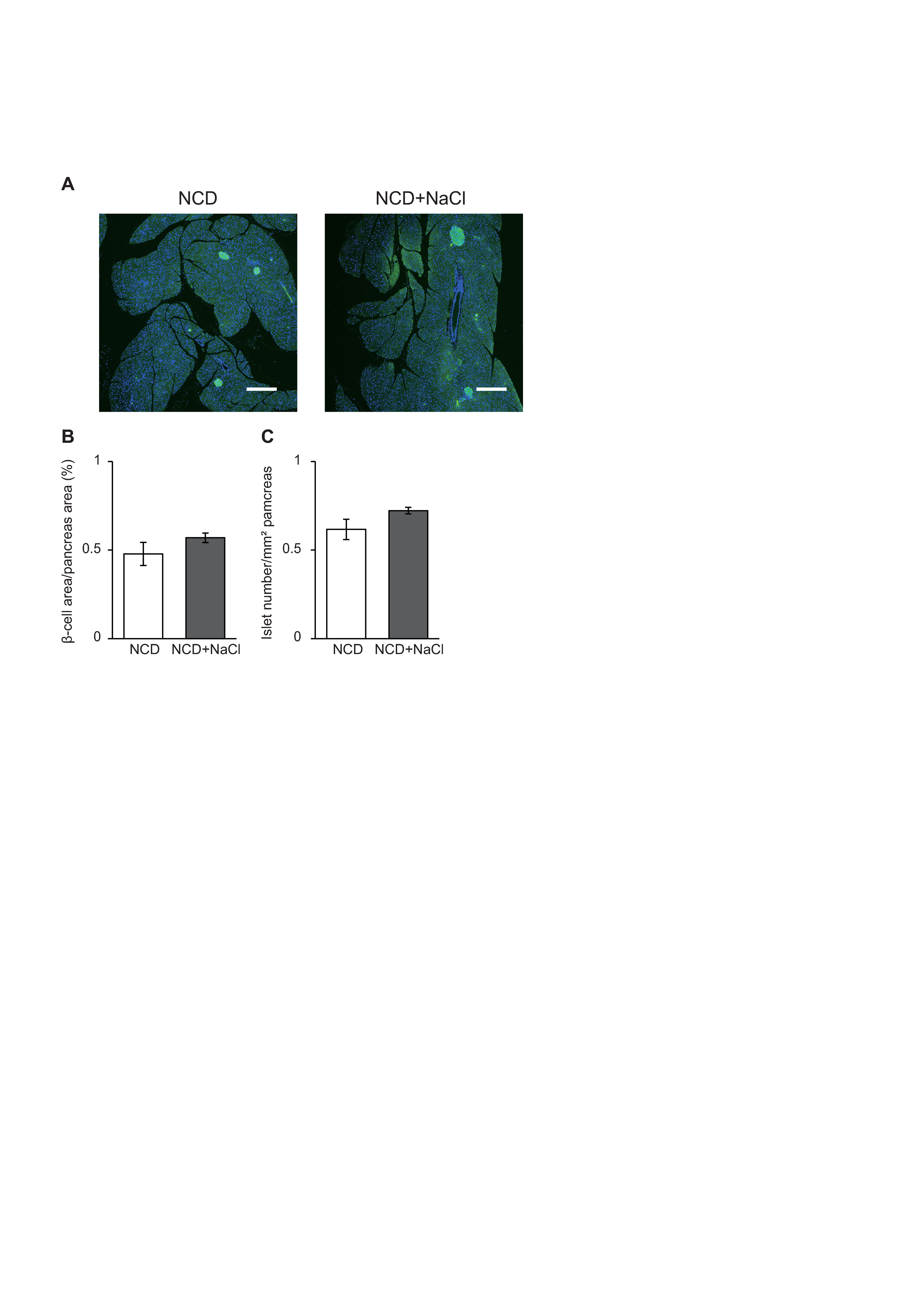

Supplement: S5 Fig — (A) Pancreatic sections stained with antibody to insulin (green) and DAPI (blue) in mice fed a normal chow diet (NCD) or NCD plus NaCl for 30 weeks. Scale bars, 100 μm. (B) Beta-cell area relative to pancreas area, and (C) the number of islets relative to pancreas area in mice fed a NCD or NCD plus NaCl for 30 weeks (n = 6/group). All values are mean ± SEM. (TIF) [file pone.0248065.s005.tif]

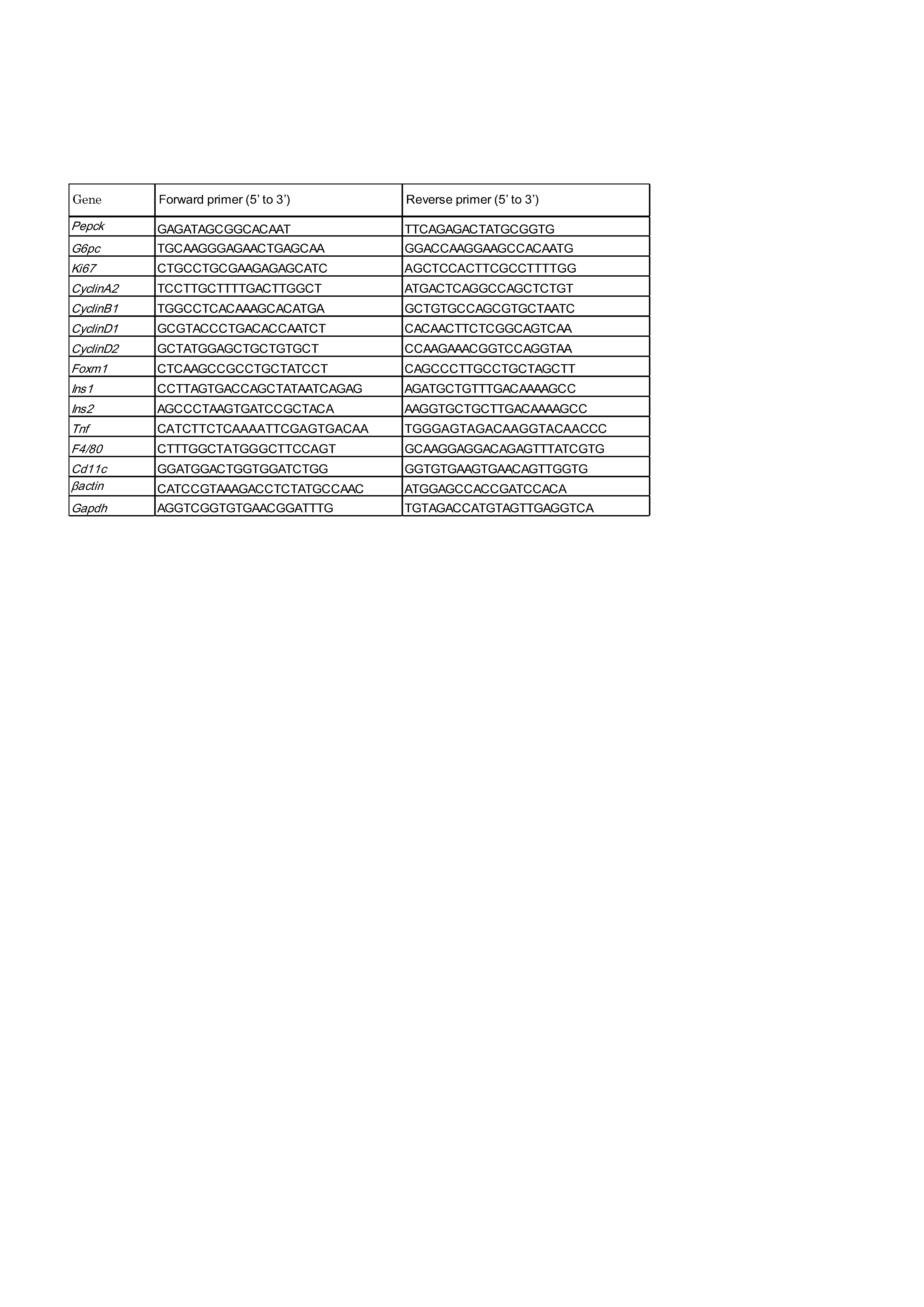

Supplement: S1 Table — (TIF) [file pone.0248065.s006.tif]

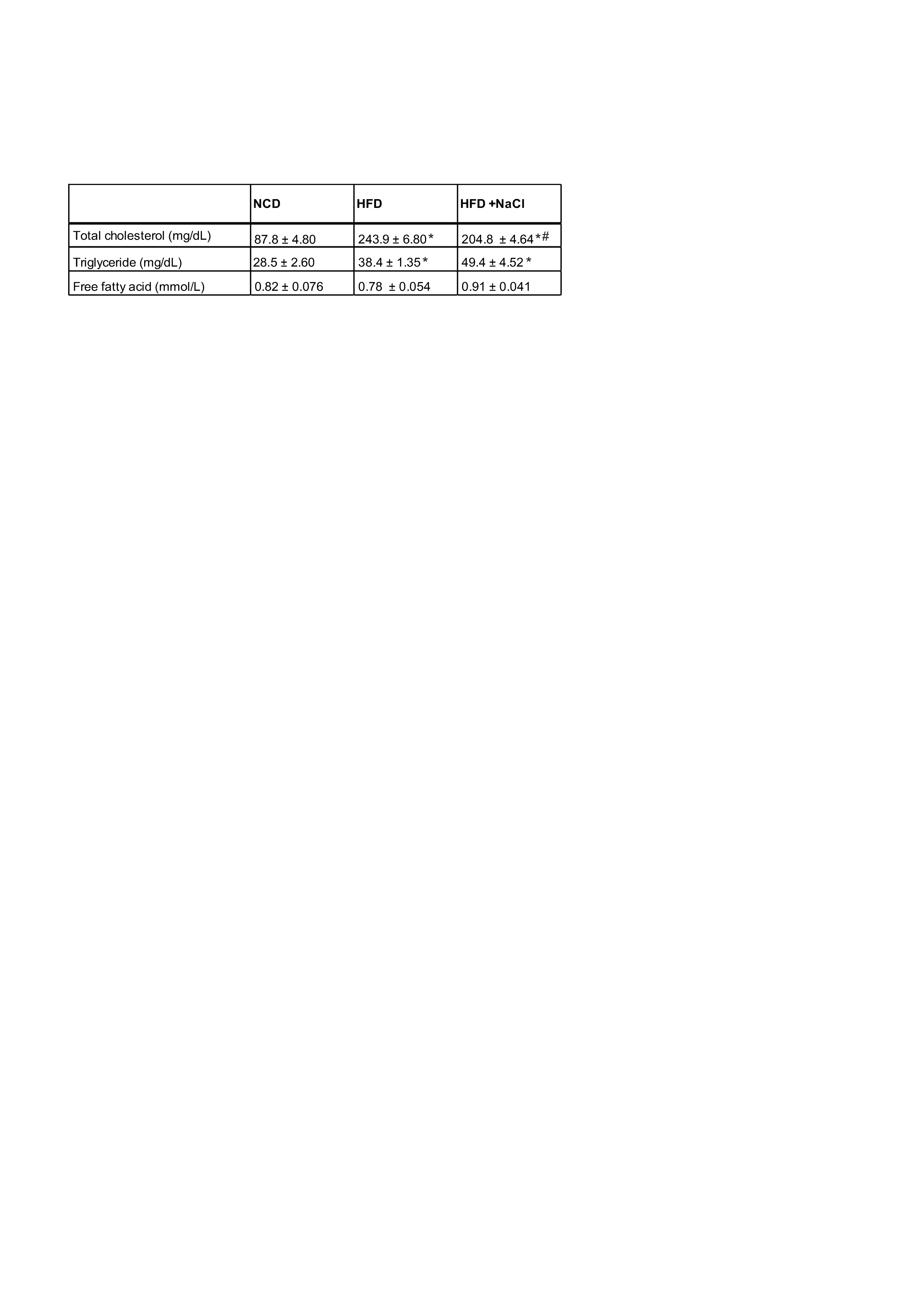

Supplement: S2 Table — Serum total cholesterol, triglyceride and free fatty acid levels in mice fed a normal chow diet (NCD), high-fat diet (HFD) or HFD plus NaCl (n = 8/group). All values are mean ± SEM. *p < 0.05 versus mice fed a NCD. #p < 0.05 versus mice fed a HFD. (TIF) [file pone.0248065.s007.tif]
