# Supplementary material for: Dietary sodium chloride attenuates increased β-cell mass to cause glucose intolerance in mice under a high-fat diet
Source: PLoS One. 2021 Mar 17;16(3):e0248065. doi: 10.1371/journal.pone.0248065 (PMC7968668; doi:10.1371/journal.pone.0248065)
Supplement: Attachment — Submitted filename: Response to Reviewers.docx [file pone.0248065.s008.docx]

**Response to Reviewers**

PONE-D-20-36592

Dietary sodium chloride attenuates increased β-cell mass to cause glucose intolerance in mice under a high-fat diet

Journal requirements:

1. Please ensure that your manuscript meets PLOS ONE's style requirements, including those for file naming. The PLOS ONE style templates can be found at

https://journals.plos.org/plosone/s/file?id=wjVg/PLOSOne_formatting_sample_main_body.pdf and

https://journals.plos.org/plosone/s/file?id=ba62/PLOSOne_formatting_sample_title_authors_affiliations.pdf

We have checked our manuscript, including file naming, carefully and confirmed that journal’s requirements are fulfilled.

2. To comply with PLOS ONE submissions requirements, in your Methods section, please provide additional information regarding the experiments involving animals and ensure you have included details on (1) methods of sacrifice, (2) methods of anesthesia and/or analgesia, and (3) efforts to alleviate suffering. Moreover, please clarify how many animals were used, and how they were assigned to the different experimental groups.

We have added the numbers of animals used in each group and the required information in the relevant subsection of the Materials and Methods (page 5, lines 76–77).

Reviewers' comments:

Reviewer #1: This manuscript has an important finding and scientific merit. Although this manuscript is well written, I have some concerns:

1) Are there four figures? The figure numbers are confusing. The manuscript shows four figures in the text but there are more and more attached figures. Also, there is a lot of supporting S figures.

There are four figures and five supplemental figures. We have indicated the figure number on each figure and submitted supplemental figures and tables as Supporting Information.

2) The Area Under the Curve (AUC) is derived from the Oral Glucose Tolerance Test (OGTT) which is widely used to diagnose the Impaired Glucose Tolerance (IGT) in the clinic. This expression is not explained in the manuscript. The journal is interdisciplinary and this term should be explained to readers.

Based on the comment, we have added an explanation in the revised manuscript (page 6, lines 100–101 and 105–106).

3) What is the mortality rate of animals? Why mice but nor rats?

We did not observe any unexpected deaths of mice during the experiments, and have commented on this point in the revised manuscript (page 10, line 170). Please understand that it is difficult to perform experiments with rats as space within our animal facility is limited.

4) It is recommended that the expression "HFD + NaCl-fed mice" is replaced by "HFD & NaCl-fed mice" or "HFD plus NaCl-fed mice" in the text.

However, the discussion is impressive and the conclusion is clear.

According to the advice given, we have changed the expression “HFD + NaCl-fed” to “HFD plus NaCl-fed mice” in the revised manuscript.

Reviewer #2: Authors demonstrated here that a combination of HF and high NaCl caused attenuation of increase in beta-cell mass, resulting in glucose intolerance. These findings are very interesting and valuable. Experimental design is also excellent.

However, authors should show blood parameters such as serum levels of free fatty acids and TG in addition to glucose. This is because these parameters are important as basic information of animal experiments in metabolism research.

Based on the advice given, we present data on serum levels of total cholesterol, triglycerides and free fatty acid, as well as an interpretation of results in the revised manuscript (page 11, lines 188-192, page 17, lines 294–297, and S2 Table).
